# DNA Authentication and Chemical Analysis of *Psilocybe* Mushrooms Reveal Widespread Misdeterminations in Fungaria and Inconsistencies in Metabolites

**DOI:** 10.1128/aem.01498-22

**Published:** 2022-11-29

**Authors:** Alexander J. Bradshaw, Talia A. Backman, Virginia Ramírez-Cruz, Dale L. Forrister, Jaclyn M. Winter, Laura Guzmán-Dávalos, Giuliana Furci, Paul Stamets, Bryn T. M. Dentinger

**Affiliations:** a School of Biological Sciences, University of Utah, Salt Lake City, Utah, USA; b Natural History Museum of Utah, University of Utah, Salt Lake City, Utah, USA; c Department of Medicinal Chemistry, University of Utah, Salt Lake City, Utah, USA; d CONACYT-UdeG, Departamento de Botánica y Zoología, Universidad de Guadalajara, Zapopan, Jalisco, Mexico; e Departamento de Botánica y Zoología, Universidad de Guadalajara, Zapopan, Jalisco, Mexico; f Fungi Foundation, Brooklyn, New York, USA; g Fungi Perfecti LLC Laboratories, Shelton, Washington, USA; Royal Botanic Gardens

**Keywords:** mycology, *Psilocybe*, fungaria, metabolomics

## Abstract

The mushroom genus *Psilocybe* is best known as the core group of psychoactive mushrooms, yet basic information on their diversity, taxonomy, chemistry, and general biology is still largely lacking. In this study, we reexamined 94 *Psilocybe* fungarium specimens, representing 18 species, by DNA barcoding, evaluated the stability of psilocybin, psilocin, and their related tryptamine alkaloids in 25 specimens across the most commonly vouchered species (Psilocybe cubensis, Psilocybe cyanescens, and Psilocybe semilanceata), and explored the metabolome of cultivated *P. cubensis*. Our data show that, apart from a few well-known species, the taxonomic accuracy of specimen determinations is largely unreliable, even at the genus level. A substantial quantity of poor-quality and mislabeled sequence data in public repositories, as well as a paucity of sequences derived from types, further exacerbates the problem. Our data also support taxon- and time-dependent decay of psilocybin and psilocin, with some specimens having no detectable quantities of them. We also show that the *P. cubensis* metabolome possibly contains thousands of uncharacterized compounds, at least some of which may be bioactive. Taken together, our study undermines commonly held assumptions about the accuracy of names and presence of controlled substances in fungarium specimens identified as *Psilocybe* spp. and reveals that our understanding of the chemical diversity of these mushrooms is largely incomplete. These results have broader implications for regulatory policies pertaining to the storage and sharing of fungarium specimens as well as the use of psychoactive mushrooms for recreation and therapy.

**IMPORTANCE** The therapeutic use of psilocybin, the active ingredient in “magic mushrooms,” is revolutionizing mental health care for a number of conditions, including depression, posttraumatic stress disorder (PTSD), and end-of-life care. This has spotlighted the current state of knowledge of psilocybin, including the organisms that endogenously produce it. However, because of international regulation of psilocybin as a controlled substance (often included on the same list as cocaine and heroin), basic research has lagged far behind. Our study highlights how the poor state of knowledge of even the most fundamental scientific information can impact the use of psilocybin-containing mushrooms for recreational or therapeutic applications and undermines critical assumptions that underpin their regulation by legal authorities. Our study shows that currently available chemical studies are mainly inaccurate, irreproducible, and inconsistent, that there exists a high rate of misidentification in museum collections and public databases rendering even names unreliable, and that the concentration of psilocybin and its tryptamine derivatives in three of the most commonly collected *Psilocybe* species (*P. cubensis*, *P. cyanescens*, and *P. semilanceata*) is highly variable and unstable in museum specimens spanning multiple decades, and our study generates the first-ever insight into the highly complex and largely uncharacterized metabolomic profile for the most commonly cultivated magic mushroom, *P. cubensis*.

## INTRODUCTION

Mushrooms belonging to the genus *Psilocybe* (Fr.) P. Kumm. have become a major scientific and public curiosity since 1957 when R. Gordon Wasson published a popular account of his first-hand experience with psychedelic mushrooms in *Life* magazine (1). Although species of *Psilocybe* (the first of which was originally described as Agaricus semilanceatus Fr.) have been known to the scientific community since the early 19th century (2), Wasson’s article was the first to make their psychoactive properties widely known. However, the ceremonial use of the psychoactive mushrooms has been practiced by the indigenous peoples of Mesoamerica for centuries.

The use of psychoactive mushrooms is reportedly depicted in petroglyphs and murals found in Siberia, Africa, and Spain, demonstrating a long-held interest in these organisms across the history of humankind (3). Further, in the 16th century, the Spanish Franciscan Friar Bernardino de Sahagún was the first European to document the ceremonial use of psychoactive mushrooms in Mexico (4–7). Sahagún, having spent 50 years there, began studying the language and culture of the Aztec, and much of this work was published in his *Historia general de las Cosas de la Nueva España*, in which he refers to psychoactive mushrooms as “teonanácatl” (later updated to “teotlaquilnanácatl” as the correct Nahuatl word), meaning “God’s flesh,” the sacred mushrooms of Mesoamerica. While being first recorded by Europeans and Western scientists in the Nahuatl population, ritual usage of psychoactive mushrooms has occurred in indigenous Mexican cultures including the Chatins, Chinantecs, Matlazincs, Mazatecs, Mixes, Purepechs, Totonacs, and Zapotecs, with names for these mushrooms specific to each culture (7, 8).

Despite psychoactive mushrooms being known and used in Mesoamerica for centuries, the publicity from Wasson’s article spurred widespread use of so-called “magic mushrooms” as recreational drugs during the psychedelic era of the 1960s and 1970s, culminating in the criminalization of psilocybin and its analogs (and by extension the organisms that produce them) as part of the “war on drugs” promulgated by Richard Nixon in 1971 (9). Based on assumptions about the universal presence of these chemicals in all *Psilocybe* spp., it soon became illegal to possess putative psilocybin-containing mushrooms in numerous countries, hobbling research on their diversity, biology, and unique psychoactive properties (10).

Recently, a renewed interest in psilocybin and *Psilocybe* spp. has emerged due to increasing evidence that psilocybin is highly effective in treating a variety of mental health problems, including anxiety and depression (11–17). This has led to a resurgence in clinical studies, many of which have demonstrated the therapeutic efficacy of psilocybin (18), as well as basic research that led to the identification of the enzymes and underlying genes responsible for psilocybin and psilocin biosynthesis (19, 20). Despite these recent advances, fundamental knowledge of *Psilocybe*, including its diversity, distribution, taxonomy, ecology, and chemistry, remains lacking or in dire need of update. In fact, even the functional role of psilocybin and psilocin under natural conditions remains a mystery (21, 22).

The genus *Psilocybe* sensu lato has been estimated to contain between 277 and 326 known species (23–25), of which 144 exhibit a characteristic inducible “bluing” reaction when damaged. This blue pigment is caused by the enzymatic oligomerization of psilocin and indicates the presence of the psychoactive compounds psilocybin and psilocin (22–24). It is now accepted that most of the non-bluing species likely belong in the genus *Deconica* (W.G. Sm.) P. Karst., and none of them are known to synthesize psilocybin or psilocin (26, 27), and yet the transfer of these species from *Psilocybe* is largely incomplete (28–32) (see Supplementary Information 1). In addition to the ongoing refinement of *Psilocybe* taxonomy, many species are considered rare, with many having been collected only once, leading to poor specimen representation in fungaria (equal to herbaria for *Fungi*; Supplementary Information 1) (33–35). This makes identification difficult without highly detailed and complete taxonomic keys (30, 36, 37) or impossible because of the dearth of specimens for authentication and poor representation in DNA sequence databases. This situation is not uncommon in mycology, where characters useful for field identification are often insufficient, resulting in rampant misidentification that cannot be resolved without molecular data (38, 39). On top of this, ambiguities about legal requirements in several countries for their storage, care, and sharing exacerbate the problem by further suppressing research, creating a challenging situation for researchers and legal authorities alike.

Although *Psilocybe* specimens may contain controlled substances, no evidence exists on their consistency, and psilocybin has been empirically identified in only ~45 accepted *Psilocybe* spp., which are only 32% of the ~140 putative bluing species (26, 35, 40, 41). Moreover, even for the species examined, the accuracy and reliability of these reports are dubious due to a lack of standards for taxonomic identification (including deposition of vouchers to allow for reexamination), lack of peer review for some studies, and inconsistency of methods used for chemical analysis (see Table S1 in the supplemental material) (40, 42). In addition, no data on the preservation of psilocybin/psilocin in fungarium specimens exist, further obfuscating if and to what degree current regulation of controlled substances applies to them, especially if these compounds actively degrade or have become unmeasurable during storage. This absence of a common set of methods, the almost complete lack of voucher-based studies, and the deficiency of information on the resilience of the controlled substances under standard fungarium storage conditions render existing information unreliable and highly suspect, with the result that there is actually little authoritative information on the chemistry of most *Psilocybe* spp.

*Psilocybe* mushrooms may produce a diversity of metabolites in addition to psilocybin and psilocin, including compounds such as bioactive β-carbolines in Psilocybe cubensis (Earle) Singer and Psilocybe mexicana R. Heim that may provide monoamine oxidase-inhibiting roles and contribute synergistically to the activity of psilocybin (43). Yet, much of the metabolome for most mushroom species remains uncharacterized. The metabolomes of a few *Psilocybe* species have been investigated recently, and genetic evidence for the biosynthesis of additional bioactive compounds was reported (44). Thus, the potential for other bioactive compounds within the metabolome of *Psilocybe* spp. to have synergistic effects when the mushrooms are consumed as whole organisms is very real, and this information will be important for potential future implementation of whole-organism-based therapies, such as those being developed in Oregon, USA (14, 45). A more comprehensive view of the metabolome of *Psilocybe* spp. is therefore urgently needed to mitigate any potential unanticipated bioactivity or adverse side effects of whole-organism consumption, as well as to provide a baseline for understanding the possible benefits of a whole-organism approach over psilocybin as a sole therapeutic.

In this study, we aimed to establish a baseline of current knowledge of *Psilocybe* identity and chemistry in public repositories, including vouchered specimens, DNA sequence databases, and the published literature. We then set out to provide an update to this knowledge with new insights on the chemical stability of psilocybin and its tryptamine intermediates in collections over time and to acquire a bird’s-eye view of the metabolome of the most widely cultivated psychedelic mushroom, *P. cubensis*.

## RESULTS

### Fungarium specimens and DNA extraction.

We curated a database of *Psilocybe* museum voucher records utilizing MycoPortal, the most inclusive online aggregator of fungal voucher specimen records (see online supplemental data) (46). After filtering out observations (which are mostly not linked to a physical voucher; *n* = 7,462) and non-*Psilocybe* species under older names, as well as reconciling synonyms, the final database consisted of 3,707 records of physical vouchers across 72 institutions found on MycoPortal. Many institutions either could not or refused to provide loan material due to the legal ambiguity of *Psilocybe* specimens. Despite the large number of vouchers present across these institutions, approximately one-third were identified only to genus level (1,151/3,707). Most of the specimens identified to species level were dominated by a few well-known taxa, i.e., *P. cubensis* (319/3,707), Psilocybe cyanescens Wakef. (100/3,707), and Psilocybe semilanceata (Fr.) P. Kumm. (138/3,707). Of the ~3,700 specimens requested, ~1,200 were received from 35 institutions.

A total of 18 species names across 94 specimens were used for DNA barcoding. Across all samples, DNA extractions yielded 5.5 to 166.1 ng/μL with 260:230 values between 0.22 and 6.52 and with 260:280 values ranging from 1.29 to 2.04 (see Table S2 in the supplemental material). Our best extraction results usually came from larger material inputs between 10 and 15 mg.

### Phylogenetic analysis.

In our analysis of all publicly available sequences identified as *Psilocybe* spp. from the National Center for Biotechnology Information (NCBI), we found a large number of likely incorrect or inconsistent names and sequences that of were very poor quality or that are highly divergent from mushroom-forming fungi. Four hundred twenty-three internal transcribed spacer (ITS) sequences in NCBI were labeled as *Psilocybe* or *Deconica* and other labels (when *Psilocybe* was included as a name in the sequence record), including one sequence labeled Stropharia rugosoannulata Farl. ex Murrill and one sequence labeled “Uncultured fungus.” Thirty-two of 39 sequences (82%) labeled as *Deconica* had specific epithets (9 species names), and six did not. Three hundred sixteen of 382 sequences (83%) labeled as *Psilocybe* had specific epithets (69 species names), and 66 did not. The most abundant *Psilocybe* species labels were *P. cubensis* (31 sequences; 10%), *P. cyanescens* (25 sequences; 8%), and *P. semilanceata* (20 sequences; 6%). Fifty-seven sequences labeled as *Psilocybe* were attributable to *Deconica* (Fig. S1).

A number of erroneous sequences were encountered in the NCBI database that will require either removal due to poor sequence quality or artifacts, or updates to the sequence identity. Some of these (MN510675 to MN510688 and MG969992) had many ambiguous bases and potential errors that introduced excessive numbers of autapomorphies, leading to exceedingly long branches in the phylogenetic tree. Two sequences labeled as *Psilocybe cubensis* (ON415277 and ON415278) are instead Fusarium spp. (pathogenic fungi distantly related to mushrooms) based on BLAST results. One sequence (DQ900972) from an environmental barcoding study (47) appears to be chimeric, with some portions of the sequence potentially being random and other parts matching *Amoebozoa* rather than *Fungi* based on BLAST. These sequences contribute nothing to species identification and are likely only to add more confusion, and we recommend removing them from the public records. One sequence labeled Psilocybe montana (Pers.) Kumm. (which should be updated to the current name Deconica montana (Pers.) P. D. Orton; AY129360) clustered with *P. cubensis*. Sequences labeled as *P. cubensis* were recovered in six clades. Some of these were other species of *Psilocybe* (e.g., *P. mexicana* and P. chuxiongensis T. Ma & K.D. Hyde) whereas others belong to *Deconica* (AY129351) or *Galerina* (AY281023). A specimen from the Universidad de Guadalajara (IBUG) identified as *Psilocybe cubensis* that was newly sequenced clustered with sequences labeled as *Stropharia rugosoannulata*. Similar to sequences labeled as *P. cubensis*, sequences labeled as *P. cyanescens* were recovered in three clades, two of which correspond to *Panaeolus* (EU029946) and *Deconica* (MF467896). Fifty-five sequences labeled as *Psilocybe* (including labels with specific epithets, no epithets, and uncultured) clustered with sequences labeled as *Deconica*.

Some phylogenetic relationships between *Psilocybe* species are recovered for the first time. Sequences of *P. cubensis* were most closely related to a sequence labeled as Psilocybe natalensis Gartz, D.A. Reid, M.T. Sm. & Eicker (89% rapid bootstrap support [BS]), together sharing a most recent common ancestor (MRCA) with *P. chuxiongensis* (100% BS) (Fig. S1). However, the sequence labeled as *P. natalensis* in GenBank (OK491080) is not derived from the type and is of uncertain provenance. The GenBank record refers to an observation on an online forum (https://mushroomobserver.org/472561) and lists the sequence isolation source as “Cultivated from a spore print” and the country of origin as “South Africa,” but no voucher specimen is indicated, and it is unclear if the data are reproducible or the source can be reexamined. Overall, the inclusion of NCBI sequences resulted in a large amount of noise in our analysis, which intensified confusion and contributed to a severe lack of clarity in sequence-based identification of *Psilocybe* spp.

**FIG 1:**
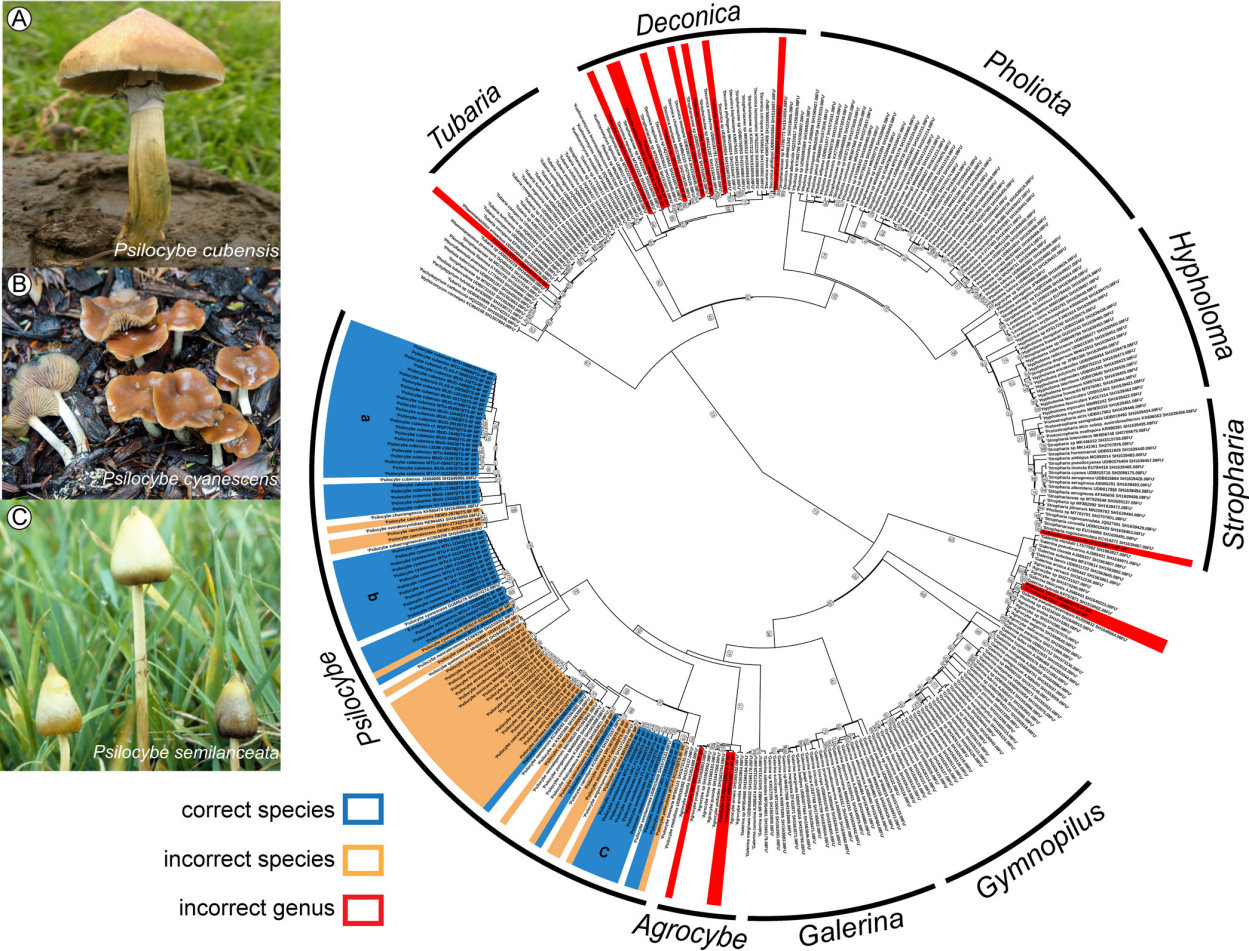
Phylogenetic tree of *Strophariaceae* and *Psilocybe.* (Right) Phylogenetic tree of museum specimen ITS sequences along with all species hypothesis (SH) sequences for the family *Strophariaceae* and the genera *Agrocybe* and *Galerina* from the UNITE fungal sequence database. Correct identifications within the genus *Psilocybe* are highlighted in blue, misidentifications at the genus level are highlighted in red, and misidentifications at the species level are highlighted in orange. (Left) Visual representation of the three main species used for this study, *Psilocybe cubensis* (photo provided by F. Landeros) (A), *Psilocybe cyanescens* (photo by B. Dentinger) (B), and *Psilocybe semilanceata* (photo by P. Stamets) (C).

Due to the large number of misidentifications and the noise in our *Psilocybe*-only analysis, we performed a second phylogenetic analysis utilizing species hypothesis (SH) sequences from the family *Strophariaceae* and the closely related genera *Gymnopilus* P. Kumm. and *Agrocybe* Fayod from UNITE (48). Of the 94 new ITS sequences generated, five have no comparable SH in the UNITE fungal ITS database (Table S2). Only 48 unique SH sequences were found in UNITE: 13 lacked species-level identification, and two are incorrectly assigned (Fig. 1). Like the NCBI records, the UNITE database also includes outdated taxonomic names such as Psilocybe coprophila (Bull.) P. Kumm. and Psilocybe subviscida (Peck) Kauffman, both of which belong to the nonpsychoactive genus *Deconica*.

Our phylogenetic analysis revealed *Psilocybe* sensu stricto to be a monophyletic group (98% BS) sharing a most recent common ancestor with species of the genus *Agrocybe* (Fig. 1), in contrast with previous work that reported other members of *Strophariaceae* as related taxa (49). Approximately 31 moderately to highly supported clades (i.e., species) of *Psilocybe* are currently represented by ITS sequences. Thirteen of the 94 specimens newly sequenced and two SH sequences from UNITE that had been identified as *Psilocybe* clustered outside the *Psilocybe* sensu stricto clade. These specimens were phylogenetically placed within clades corresponding to the genera *Agrocybe*, *Deconica*, *Galerina* Earle, *Stropharia* (Fr.) Quél., and *Tubaria* (W.G. Sm.) Gillet. Additionally, multiple groupings within *Psilocybe* had incongruent taxonomic names, which suggests either misidentification or possible synonyms (Fig. 1).

Although taxonomic inconsistencies occurred in every clade, some clades contained more divergent taxonomic names than others. Groupings within the sister clade to *Tubaria* included eight different generic or family-level-only identifications [*Pachylepyrium* Singer, *Phaeomarasmius* Scherff., *Pholiota* (Fr.) P. Kumm., *Pleuroflammula* Singer, *Psilocybe*, *Mythicomyces* Redhead & A.H. Sm., *Strophariaceae*, and *Tubaria*]. Additionally, the branch belonging to *Deconica* included two SH sequences attributed to *Psilocybe*, six of our vouchered specimens, and eight SH sequences labeled *Strophariaceae* species SH sequences (Fig. 1). *Agrocybe* and *Galerina* were recovered as paraphyletic, with a single branch (63% BS) that also included two of our vouchered specimens labeled as *Psilocybe* and SH sequences labeled as *Pholiota* spp.

### Tryptamine metabolite profiling in voucher specimens.

**FIG 2:**
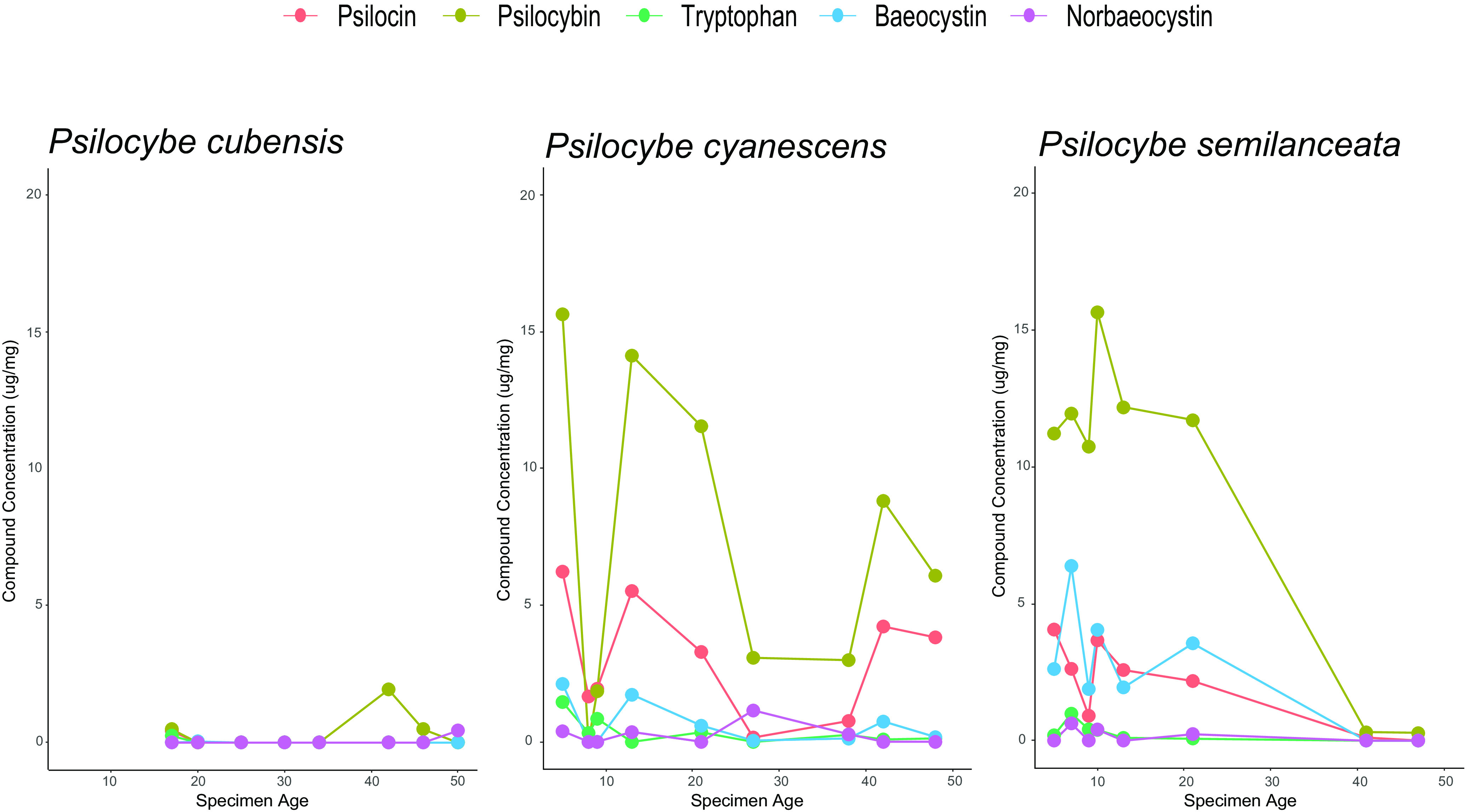
Content of tryptophan, psilocybin, and related alkaloids in fungarium specimens of three species of *Psilocybe* over 5 decades. Species are *Psilocybe cubensis* (left), *Psilocybe cyanescens* (center), and *Psilocybe semilanceata* (right). Specimen age (years) is on the *x* axis, and concentration of metabolites is on the *y* axis in micrograms per milligrams (dry weight). Points indicate concentrations of individual compounds: tryptophan (green), baeocystin (blue), norbaeocystin (purple), psilocybin (yellow-green), and psilocin (red).

We chose specimens of the three most commonly vouchered species (*P. cubensis*, *P. cyanescens*, and *P. semilanceata*) for chemical analysis and taxonomically verified each using full-length ITS barcoding that corresponded to the SH for their respective voucher identification. A single specimen of *P. cubensis* was misidentified (IBUG-10258, equals *Stropharia rugosoannulata*), and one specimen of *P. cyanescens* was misidentified (SFSU-F-029946, equals Psilocybe allenii Borov., Rockefeller & P.G. Werner) (Table 1 and Table S2). The quantities of all metabolites analyzed varied substantially between species (Fig. 2). Very few fungarium specimens of *P. cubensis* showed detectable levels of psilocybin or psilocin compared to most dried specimens of *P. cyanescens*, which had detectable levels of both compounds, albeit reduced, even after nearly 50 years of storage (Fig. 2).

**TABLE 1:** Specimen voucher table of *Psilocybe* used for chemical analysis

Institution code^*a*^	Accession no.	Voucher taxonomic designation	Collection yr
WTU	WTU-F-054871	*Psilocybe cubensis*	1970
WTU	WTU-F-011258	*Psilocybe cubensis*	1974
WTU	WTU-F-054869	*Psilocybe cubensis*	1978
IBUG	2924	*Psilocybe cubensis*	1986
IBUG	13422	*Psilocybe cubensis*	1990
IBUG	14027	*Psilocybe cubensis*	1995
IBUG	11267	*Psilocybe cubensis*	2000
NY	1901145	*Psilocybe cubensis*	2003
WTU	WTU-F-011538	*Psilocybe cyanescens*	1972
WTU	WTU-F-011519	*Psilocybe cyanescens*	1978
WTU	WTU-F-011079	*Psilocybe cyanescens*	1982
SFSU	SFSU-F-029949	*Psilocybe cyanescens*	1993
WTU	WTU-F-011523	*Psilocybe cyanescens*	1999
SFSU	SFSU-F-029967	*Psilocybe cyanescens*	2007
UBC	F24013	*Psilocybe cyanescens*	2011
UBC	F30955	*Psilocybe cyanescens*	2012
UBC	F32228	*Psilocybe cyanescens*	2015
SFSU	SFSU-F-029972	*Psilocybe semilanceata*	1973
WTU	WTU-F-055015	*Psilocybe semilanceata*	1979
UBC	F15293	*Psilocybe semilanceata*	1999
UBC	F17025	*Psilocybe semilanceata*	2007
UBC	F22104	*Psilocybe semilanceata*	2010
UCSC	UCSC-F-00856	*Psilocybe semilanceata*	2011
UBC	F32307	*Psilocybe semilanceata*	2013
FLAS	FLAS-F-63134	*Psilocybe semilanceata*	2015

a WTU, University of Washington Herbarium; IBUG, Universidad de Guadalajara; NY, New York Botanical Garden; SFSU, Harry D. Thiers Herbarium, San Francisco State University; UBC, University of British Columbia Herbarium; UCSC, University of California, Santa Cruz; FLAS, University of Florida Herbarium.

Concentrations of psilocybin and psilocin, when detectable at all, varied in *P. cubensis* voucher specimens from 0.55 to 1.9 μg/mg (dry weight), with only a single sample having detectable amounts of psilocin at 0.44 μg/mg (dry weight). *Psilocybe cyanescens* specimens contained relatively more and consistently higher alkaloid concentrations, with psilocybin content ranging from 3.0 to 15.6 μg/mg (dry weight) and psilocin content ranging from 0.22 to 5.22 μg/mg (dry weight). *Psilocybe semilanceata* specimens contained psilocybin content ranging from 0.33 to 15.77 μg/mg (dry weight) and psilocin content ranging from 0.1 to 3.88 μg/mg (dry weight) (Fig. 2). Specimen age was poorly correlated with quantity of alkaloids. Alkaloids were low or nearly undetectable in some specimens of *P. cubensis* and *P. cyanescens* <20 years old but also detectable at multiple-microgram-per-milligram concentrations in nearly 50-year-old specimens of *P. cyanescens*. Older specimens (>20 years) of *P. semilanceata* had barely detectable levels of the indole alkaloids.

### Psilocybin and psilocin metabolite content in cultivated *P. cubensis*.

Psilocybin was the major alkaloid recovered from mycelium (6.44 μg/mg), caps (10.5 to 20 μg/mg), and stipes (15.44 to 18.44 μg/mg) (Table 2). Psilocin was a minor component in all samples (0.44 to 2.0 μg/mg). In comparison, psilocybin-to-psilocin ratios ranged from ~5:1 to 47:1 in the caps and ~7.5:1 to 37:1 in the stipes (Table 2). Performing an unpaired *t* test, we found no significant difference in psilocybin or psilocin between whole individual sporocarps (Psi 5 and Psi 6) from the same reproductive event in a single growth bin (psilocybin percentage point [pp] = 0.1349 and psilocin pp = 0.4756). Unpaired *t* tests also showed no significant difference in concentrations between the same anatomical tissues (caps and stipes) or between the two sporocarps Psi 5 and Psi 6 (psilocybin pp = 0.5081 and psilocin pp = 0.4563) or measured across Psi 4, Psi 5, and Psi 6 (psilocybin pp = 0.8182 and psilocin pp = 0.6898). However, at the full sporocarp level, quantities of psilocybin and psilocin were statistically different between growth bins (psilocybin pp = 0.0560 and psilocin pp = 0.0389).

**TABLE 2:** Psilocybin and psilocin concentrations in cultivated sporocarps of *Psilocybe cubensis*

Sample identifier	UT-M catalog no.	Amt (mg) sampled of voucher specimen	Psilocybin concn (μg/mg)	Psilocin concn (μg/mg)	Source (origin of sample)
Psi-mycelium	UT-M0001771	7	6.3	0.01	Stock mycelium culture
Psi-4-Cap	UT-M0001772	7	10.5	2.11	Single sporocarp from one growth bin
Psi-4-Stipe	UT-M0001772	8	15.44	1.9	Single sporocarp from one growth bin
Psi-5-Cap	UT-M0001773	7	17.3	0.5	One of two sporocarps from one growth bin
Psi-5-Stipe	UT-M0001773	9	16.33	0.4	One of two sporocarps from one growth bin
Psi-6-Cap	UT-M0001774	9	19.9	0.44	Two of two sporocarps from one growth bin
Psi-6-Stipe	UT-M0001774	7	18.3	1.5	Two of two sporocarps from one growth bin

### Metabolomic profiling and tissue comparison of cultivated material.

**FIG 3:**
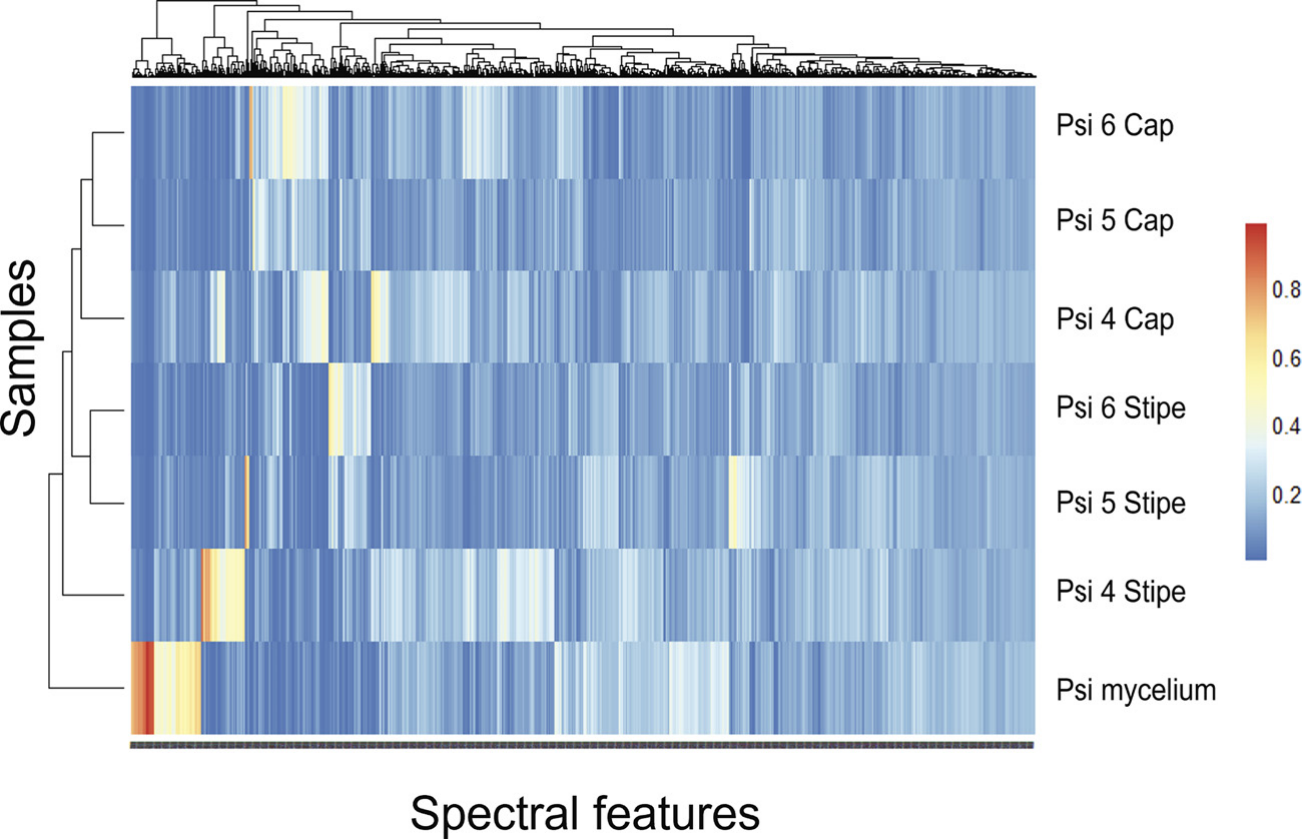
Comparison of chemical profiles across cultivated *Psilocybe cubensis* samples. Three sporocarps and one mycelium were used for chemical profiling. Sporocarps were harvested simultaneously from the same growth bin (Psi 5 and 6) and from a second growth bin (Psi 4) grown from a single mycelial culture. Hierarchical clustering of samples (*y* axis, left) and spectra (*x* axis, top) based on Euclidean distance similarities of total chemical profile and spectral feature similarity, respectively. Heatmap shows chemical factor contribution using the intensity values for each identified factor represented by a color gradient from blue (low contribution) to red (high contribution).

We identified 1,465 distinct spectral features based on their unique mass-to-charge (*m/z*) ratio and retention time. Using hierarchical clustering to compare the intensity profiles of each of our *P. cubensis* samples across the two growing environments, we found that metabolic profiles of caps and stipes were most like one another, with the mycelium being most different from all other samples, regardless of which container they were cultivated in (Fig. 3). However, in the case of both caps and stipes, Psi 5 and Psi 6 were shown to be more alike, while Psi 4 formed an outgroup for each tissue type (Fig. 3). We investigated this further by performing a principal-component analysis (PCA) on the intensity of each feature and partial least-squares discriminant analysis (PLS-DA) for each sample (Fig. S3 and S4). PCA showed largely overlapping spectral intensities, but the same pattern as that in the hierarchical clustering was present, with dimension 1 accounting for 15.6% of the variability and dimension 2 accounting for 63.3% of the variability (Fig. S3). PLS-DA on the full profiles for each sample, rather than the individual spectral features, again showed a pattern of caps and stipes being most similar but distinct separation between samples Psi 4 (growth bin 1) and Psi 5 and Psi 6 (growth bin 2), even though all samples were cultivated and harvested in the same manner.

In addition to looking at broad-scale patterns of metabolomic profiles, we also wished to investigate if any of the identified spectral features could be annotated to known compounds. We collected tandem mass spectrometry (MS/MS) data for sample Psi-6-Cap for comparison to the Global Natural Products Social Molecular Networking database (GNPS; https://gnps.ucsd.edu/ProteoSAFe/static/gnps-splash.jsp); (50). Using the GNPS spectral library matching the compound database provides an annotation along with a cosine score (MQscore) that indicates spectral similarity to the annotated compound. An MQscore of 1 suggests an identical match of a spectral feature to the annotated compound in the database, with decreasing scores suggesting compounds of similar structure as the annotation. Spectral library matching for Psi-6-Cap revealed 30 features that could be annotated to known compounds (0.02% of the identified 1,465 features) (Table S5). Many of these annotations belonged to compounds involved in cell membrane formations, sugars, and cholesterol (*sn*-glycero-3-phosphocholine MQscore = 0.97, sucrose MQscore = 0.93, and epicoprostanol MQscore = 0.92), which are likely to be found in high quantities because these profiles were derived from freshly preserved specimens. However, three annotated spectral features were of particular interest as they had structural similarity to serotonin (MQscore = 0.9292), cocamidopropyl betaine (CAPB) (MQscore = 0.9191), and trazadone hydrochloride (MQscore = 0.73).

## DISCUSSION

Our study shows that fungarium specimens of *Psilocybe* spp. are regularly misidentified, little has been done to investigate the stability of the known psychoactive compounds under standard storage conditions, and the chemical complexity of these organisms has been overlooked by an almost exclusive focus on the principal psychoactive agents. These deficiencies cast a shadow on what little information is available on *Psilocybe* and underscore the increasingly urgent need for comprehensive basic research on them.

### Authentication and diversity of *Psilocybe* fungarium specimens.

Fungi often exhibit cryptic morphology and can be difficult to identify, even for highly trained experts. Cryptic traits, lack of extensive training, and even clerical errors in databasing can all contribute to misidentification (38, 51). In this sense, it is likely that specimens in fungaria of nearly all taxa suffer from some level of taxonomic uncertainty, but the extent to which misidentification is a problem has been rarely investigated. A dearth of authentic DNA barcodes for *Psilocybe* and other closely related groups further exacerbates the problem. Without DNA barcodes from type or other authentic material, names can be applied inconsistently, leading to different applications of species names to modern collections. This also makes it difficult to know when a specimen represents a new species, resulting in further misapplication of named species to novel taxa. We found that close to 13% of the specimens we studied that were cataloged as *Psilocybe* belonged to other genera, none of which are known to contain controlled substances. This result challenges the use of names in collections for meta-analysis or evidence-based decisions on their custody and care. Many publicly available sequences are incorrectly labeled or are of very poor quality, further aggravating the problem for identifying specimens or other samples using a DNA barcoding approach. Even some of the most recognizable and frequently collected species (*P. cubensis* and *P. cyanescens*) had sequences that belonged to other related genera and in two cases to fungi from a different phylum (*Ascomycota*). Some of these are likely misidentification of the source material, including cultures, highlighting the need for an authoritative, expert-curated reference database of DNA sequences derived from types or other authentic specimens.

While misidentifications in museum collections and public DNA repositories are not uncommon (38, 39), the ambiguous legal status of *Psilocybe* specimens makes misidentifications detrimental not only to scientific research but also to properly applying regulatory policies. Misidentification of putative psilocybin-producing mushroom specimens may lead to an unnecessary effort and poor use of resources, or even miscarriage of justice in legal cases.

A review of the currently known species of *Psilocybe* suggests that 165 belong to *Psilocybe* sensu stricto while 98 belong to *Deconica* or other genera (see Supplementary Information 1 in the supplemental material). Fifty-two species that likely belong to *Deconica* are currently still classified in *Psilocybe*. However, these likely include synonyms and cryptic species that are presently unknown and do not account for undocumented species that likely exist. Only five species of *Psilocybe* sensu stricto are represented by ITS barcodes derived from types. Critically, sequences from types of the best-known species, such as *Psilocybe cubensis*, do not exist. This fundamental information is critical for accurate application of names and should be considered an urgent priority to aid in the identification of this medically and societally important group.

While our data set had limited representation of the known species of *Psilocybe*, new phylogenetic patterns were uncovered. While Ramírez-Cruz et al. (34) reported that the American species *P. cubensis* shared a most recent common ancestor (MRCA) with the southeast Asian species Psilocybe thaiaerugineomaculans Guzmán, Karun. & Ram.-Guill., our inclusion of the South African *P. natalensis* and the more recently described Asian species *P. chuxiongensis* revealed that *P. cubensis* likely shares an MRCA with *P. natalensis* and not the Asian species. This is consistent with the African-origin hypothesis for *P. cubensis* and/or its progenitor proposed by Guzmán et al. (52) and Froese et al. (53) rather than the Asian-origin hypothesis suggested by the relationships to Asian species in the work of Ramírez-Cruz et al. (34) and Ma et al. (54). However, it should be noted that the sequences of *P. natalensis* and *P. thaiaerugineomaculans* are not derived from type specimens (which is also true for *P. cubensis*), and so while the geographic implications remain, the taxonomic ones are uncertain. To gain a better understanding of the geographic origins of *Psilocybe* spp., especially the enigmatic *P. cubensis*, new fieldwork to expand documentation of *Psilocybe* in Africa and Asia should be a priority.

### Psilocybin, psilocin, and related metabolites in *Psilocybe* spp.

Decades of inconsistency in the identification of *Psilocybe* and methods for measuring its metabolites render most previous studies unreliable and unreproducible. In our review of the published literature on alkaloid chemistry in *Psilocybe*, there was an almost complete lack of thorough sample identification, few studies designated vouchers, and literature citations frequently contained incorrect information or opposing statements (see Table S1 in the supplemental material). In a few instances, reports for some species, such as Psilocybe ovoideocystidiata Guzmán & Gaines and Psilocybe kumaenorum R. Heim, lacked peer review or were not accessible. Although our focus was specifically on literature pertaining to *Psilocybe*, psilocybin has been reported from other species such as Agrocybe praecox (Pers.) Fayod (55, 56), although there is good reason to believe this may have been a misidentification of Psilocybe subcubensis Guzmán (57). Unfortunately, because no vouchers were deposited, it is impossible to reexamine the material to determine the true identity. Nonetheless, this dubious report has persisted through repetition without critical review or scrutiny of the data. A much more rigorous set of standards is needed to establish a reliable baseline for information on the chemistry of psilocybin and related alkaloids in mushrooms.

We chose to quantify psilocybin and related metabolites in fungarium specimens of *Psilocybe* to determine their presence and stability over time. One recent study showed that the method of drying and extent of light exposure can affect psilocybin/psilocin stability in *P. cubensis* (58), but this examined storage over only a single year. Our specimens from three species spanned multiple decades, and we found that metabolite content and stability varied widely and metabolite content was undetectable in most samples stored for more than 40 years (Fig. 2). However, the sample size for any given specimen age was small, and information on confounding factors, such as the original preservation method, was often not available. In one instance, we were able to measure the chemical content of a specimen of *P. cubensis* (WTU-F-054869) deposited around the same time as specimens used for previous chemical analysis (26, 59, 60). Although there is a strong possibility that this specimen was the same one used in the original study or was harvested from the same cultivated strain around the same time (M. Beug, personal communication), no voucher information was available from the original study and so it was not possible to confirm its identity. Bigwood and Beug (60) noted that multiple flushes of *P. cubensis* contained over 5.0 μg/mg (dry weight) of psilocybin. However, when we measured the WTU-F-054869 voucher, now 42 years old, we detected less than half of their reported psilocybin content (1.9 μg/mg) and no psilocin (Fig. 2). This provides further evidence that these chemicals are highly unstable in preserved specimens of *P. cubensis*, degrading either in a time-dependent manner or possibly during or soon after the initial preservation. A study designed to specifically test time-dependent degradation with replicates and controls would be needed to determine the exact causes of the metabolite instability we observed.

Our results also reveal taxon-dependent patterns of metabolite instability. For example, psilocybin and psilocin in *P. cyanescens* had a comparably lower rate of degradation over time than in *P. cubensis*, with the youngest specimen having 15.6 μg/mg psilocybin and 5.8 μg/mg psilocin and the oldest (40-year) specimen having 6.11 μg/mg psilocybin and 3.66 μg/mg psilocin. Similarly, in *P. semilanceata* the youngest sample contained 11.2 μg/mg of psilocybin and 3.88 μg/mg psilocin, while the oldest contained 0.2 μg/mg psilocybin and no detectable psilocin. One possible explanation for the generally better chemical preservation in *P. cyanescens* and *P. semilanceata* is that these species have sporocarps that are comparatively lower in biomass than those of *P. cubensis*. This may result in a more rapid desiccation during heat-assisted drying, minimizing the opportunity for spontaneous and/or enzymatic degradation of psilocybin and psilocin. Another possible explanation may be from differences in the expression or catalytic activity of the enzymes involved in conversion of psilocybin and psilocin (22). However, the overall pattern of degradation for the other metabolites we measured mirrored that of psilocybin and psilocin (Fig. 2), suggesting spontaneous rather than enzyme-assisted degradation. Nonetheless, in any given specimen, degradation patterns are likely to differ based on a complex set of factors such as age, preservation method, and species. Taken together, our results illustrate that the presence and quantities of psilocybin and psilocin in fungarium specimens are highly unpredictable.

To investigate if the initial method of specimen preservation has a large effect on psilocybin and psilocin degradation (58), we compared psilocybin and psilocin in freshly harvested specimens that were flash frozen in liquid N_2_ and lyophilized. This preservation method is thought to optimally preserve naturally occurring concentrations of psilocybin and psilocin by protecting psilocybin against hydrolytic dephosphorylation (61). Our lyophilized sporocarp samples had 10.55 to 19.9 μg/mg psilocybin and 0.44 to 2.0 μg/mg psilocin, which is consistent with previous reports, including the high ratio of psilocybin to psilocin (Table S1). These quantities are much higher than anything we detected in heat-dried fungarium specimens of *P. cubensis*, indicating lyophilization may be the best method for preserving psychoactive alkaloids in this species.

Psilocybin and psilocin concentrations have been shown to vary among mycelium and parts of the mushroom (21, 62). In an effort to document this variation in our cultivated *P. cubensis* strain, we independently analyzed psilocybin and psilocin concentrations in different samples (mycelium, stipe, and cap) from multiple sporocarps within the same flush (Psi 5 and Psi 6) and from sporocarps between two growth containers (Psi 5/Psi 6 versus Psi 4). Mycelium showed one-third the content of psilocybin and five to 20 times less psilocin than did the stipe and cap, respectively (Table 2). Although we found comparatively more alkaloids in the caps than in the stipes, the concentrations of psilocybin and psilocin at the full sporocarp level were statistically different between growth bins despite using the same mycelial stock and growth conditions. This suggests that using consistent strain and growth conditions would yield predictably similar concentrations of both psilocin and psilocybin within a growing environment and between caps and stipes but that inconspicuous microenvironmental conditions could be relevant to the metabolomic phenotype (Fig. 2 and Table 2). These results have important implications for the use of whole sporocarps for therapeutic or recreational applications, and better replicated studies are needed to elucidate the nuances of this variation.

### Untargeted metabolomics reveals a large diversity of uncharacterized metabolites in *P. cubensis*.

Although the vast majority of chemical studies of *Psilocybe* have focused on the principal psychoactive alkaloids and their intermediates, *Psilocybe* spp. almost certainly produce a diversity of other metabolites, at least some of which may be bioactive (43). Our total metabolomic profile of cultivated *P. cubensis* detected 1,465 unique spectral features that varied among tissue types and growth replicates (Fig. 3 and Fig. S3 and S4). Only 0.2% of these could be assigned to known chemicals using the GNPS reference database. While becoming more common, studies of untargeted metabolomics in fungi are relatively new and often focus on *Ascomycota* rather than *Basidiomycota* because of their historical use for antimicrobial and natural product discovery (63). The general lack of these studies makes it difficult to judge if our metabolic profile is typical of *Basidiomycota*, as some species, such as Trametes versicolor (L.) Lloyd, have been shown to produce different profiles based on the presence of other fungi (64). Developmental variation is also likely. For example, Ganoderma sichuanense J.D. Zhao & X.Q. Zhang (equals Ganoderma lingzhi Sheng H. Wu, Y. Cao & Y.C. Dai) can have as many as 9,000 spectral features that differ depending on the life cycle stage in which they are investigated (65). While undoubtedly highly variable, the metabolomic profile of our sporocarps represents a snapshot of mature *P. cubensis* mushrooms grown in a conventional medium, which may be useful for future comparison and chemical exploration of cultivated material.

Spectral features with similarity to both serotonin and trazodone hydrochloride were detected in *P. cubensis*. Serotonin is a neurotransmitter that modulates many neurological processes (66), while trazodone hydrochloride is a synthetic triazolopyridine derivative with serotonin-selective reuptake inhibition (SSRI) activity commonly prescribed as an antidepressant (67). Serotonin has been reported from *Panaeolus* (Fr.) Quél., some species of which also produce psilocybin (68, 69), and many other species of *Basidiomycota* (70). The synthetic trazodone hydrochloride is far less likely to be present in the mushrooms since it is not a natural product. However, motifs and chemical structures with similarity to synthetic compounds have been found to be naturally occurring in other fungi (71), so the possibility that some fungal natural products could mimic the known function of these synthetic compounds is not unreasonable. Even if these two compounds were incorrectly identified, these results clearly demonstrate that additional chemicals in the metabolome of *P. cubensis* have real potential to contribute to physiological and neurological activity if consumed.

In addition to endogenous metabolites, the accumulation of chemical compounds from the growth substrate is a potential source of other physiologically active metabolites. For example, we detected cocamidopropyl betaine (CAPB), a chemical component of coconut (72). CAPB is a common human irritant and allergen (72–74), and its presence in the substrate when cultivating *P. cubensis* sporocarp may be a cause for concern for some people. While we cannot absolutely confirm that this spectral feature is CAPB, neither can we rule out that this compound is endogenously produced by *P. cubensis*, as our sporocarps were produced using a casing of coconut coir fiber, a likely source of this compound. This would suggest that the accumulation of chemicals and the overall chemical composition of cultivated sporocarps may be a combination of both endogenous and exogenous sources. Indeed, mushrooms are known to sequester chemicals from the environment, where they have been used to remove toxins (75, 76). This is of particular importance for the potential therapeutic or recreational use of whole mushrooms and requires empirical data to make informed decisions on policies around their production and regulation (77).

Despite a surge in interest in *Psilocybe* due to accumulating evidence of the therapeutic potential of psilocybin, the current state of knowledge of *Psilocybe* and the psychoactive compounds it produces is still in its infancy. Our study shows that much more research is needed to achieve a more thorough, accurate, and reliable understanding of this enigmatic and socially important group to develop meaningful policies around its use and care, and improve its application for human wellbeing.

## MATERIALS AND METHODS

### Fungarium voucher authentication with DNA barcodes and phylogenetics.

**(i) Fungarium specimens.** We created a curated database of voucher information for all collections identified as the genus *Psilocybe* using MycoPortal (46) accessed on 1 June 2020. Records without physical vouchers (i.e., observations only) were removed, and the remaining names were corrected by comparing them to the most up-to-date list of currently accepted names in Species Fungorum (78). We subsampled specimens specifically for vouchers that were identified to species level and attempted to sample a minimum of three specimens with the same taxonomic name, totaling 94 specimens, to maximize the diversity of our data set for accurate species comparison (see Table S1 in the supplemental material).

**(ii) DNA extraction and barcode sequencing.** Total DNA was extracted from the hymenophore in an amount ranging from 5 to 15 mg. These fragments were ground to a fine powder by placing them in 2.0-mL screw-cap tubes containing a single 3.0-mm bead and 8- by 1.5-mm stainless steel beads and shaking them in a BeadBug microtube homogenizer (catalog no. Z763713; Sigma) for 120 s at speed setting 350. The ground sample was used as input to the Monarch genomic DNA purification kit (New England Biolabs [NEB] catalog no. T3010S) following the manufacturer’s instructions except using twice the volume of lysis buffer and increasing the amount of wash buffer to 550 μL during both of the washing steps. These samples were amplified using the PCR primers ITS-8F (5′-AGTCGTAACAAGGTTTCCGTAGGTG-3′) and ITS-6R (5′-TTCCCGCTTCACTCGCAGT-3′), which were specifically designed for Agaricomycetes (79), and submitted for Sanger sequencing on both strands through the Genewiz Corporation as well as the DNA sequencing core at the University of Utah.

Complementary sequencing chromatograms were then processed by creating a consensus sequence using Sequencher 5.4 (http://www.genecodes.com) and trimming the 5′ and 3′ ends to conserved motifs 5′-CATTA- and -GACCT-3′ following the method in reference 79 for downstream analysis.

**(iii) Phylogenetic analysis.** To validate the identities of the voucher specimens, we subjected the ITS sequences to phylogenetic analysis using two data sets. The first consisted of all currently available ITS sequences from the National Center for Biotechnology Information (NCBI) identified as *Psilocybe* spp. using the query ‘Psilocybe [ORGN] AND “internal transcribed spacer”’ (*n* = 384). The second included all sequences belonging to the Species Hypothesis (SH) (generated at the 1.5% similarity threshold) of family *Strophariaceae* Taxon Hypothesis (80) TH006803 (244 SH, 26 sequences assigned to *Psilocybe*) found in the UNITE database (UNITE classifies *Psilocybe* in *Strophariaceae*; general release, 10.05.2021) (81). Outgroup SH sequences for the genera *Agrocybe* (TH012107) and *Gymnopilus* (TH012136) were included based on phylogenetic relationships provided by JGI MycoCosm (https://mycocosm.jgi.doe.gov/mycocosm/species-tree/tree;Y-nUEB?organism=agaricales). The motivation for using the UNITE SH sequences independent of NCBI sequences is that the UNITE database is curated to minimize noise by reducing minor sequence variation using representative sequences chosen manually by expert curators or automatically using a standard dynamic similarity threshold. All SHs are assigned a unique digital object identifier (DOI) to allow stable, unambiguous reference across studies, even in the complete absence of meaningful taxonomic names (82).

For *Psilocybe*, expert verification has been completed for 9/48 SHs, for which a representative sequence has been manually chosen, whereas the remaining SHs are represented by a sequence automatically chosen by the SH algorithm.

ITS barcode sequences generated in this study were combined with data set 1 and data set 2 for phylogenetic analysis.

For all data sets, sequences were automatically aligned using the multiple sequence alignment software MAFFT v7.475 (83) with the L-INS-i algorithm followed by phylogenetic inference under maximum likelihood (ML) using IQ-TREE v22.0.3 (84) with the automatic ModelFinder setting (85) and 1,000 ultrafast bootstrap replicates (86). The best ML trees were rendered in FigTree v1.4.4 (http://tree.bio.ed.ac.uk/software/figtree/).

### Tryptamine metabolite profiling in voucher specimens.

**(i) Specimen preparation and chemical extraction.** Eight *Psilocybe cubensis* vouchers (including specimens deposited in the University of Washington Herbarium [WTU] around the same time as those analyzed in reference 26), six cultivated *P. cubensis* samples (three sporocarps split into caps [=pileus] and stipes), nine *P. cyanescens* samples, and eight *P. semilanceata* samples were used for chemical analysis across multiyear time points (*P. cubensis* = 17 to 50 years, *P. cyanescens* = 5 to 48 years, and *P. semilanceata* = 5 to 47 years). Samples were ground to a fine powder, as described above, prior to solvent extraction as described in the work of Fricke et al. (20). Briefly, homogenized samples were resuspended in a 1:20 ratio of sample (milligrams) to methanol (microliters) and then sonicated in a Branson 1510 sonicator for 30 min. The sonicated solution was then passed through a 0.22-μm filter, and the filtered solution was diluted to 100 μg/mL with a methanol-water (50:50) solution and injected on the ultrahigh-performance liquid chromatograph/mass spectrometer (UHPLC-MS).

**(ii) UHPLC-MS method to identify and quantify psilocybin and tryptamine alkaloid intermediates.** Hydrophilic interaction liquid chromatography (HILIC)-mass spectrometry was performed using an Acquity Arc UHPLC-MS (Waters, Milford, MA, USA) in both positive and negative mode with a Waters Acquity UPLC ethylene-bridged hybrid (BEH) amide column (1.7 μm, 2.1 by 100 mm) set to 40°C. The λ range was set at 200 to 500 nm with a resolution of 1.2 nm and a sampling rate of 10 points/s. The mass range in positive mode was set at 100 to 800 Da with a gain of 1, probe at 600°C, a capillary voltage of 0.8 kV, and collection at 10 points/s. A flow rate of 0.5 mL/min and a linear solvent gradient of 99.9% solvent A (10 mM ammonium acetate, 95% acetonitrile, 0.014% ammonium hydroxide, pH 9.0) to 30% solvent B (10 mM ammonium acetate with 0.014% ammonium hydroxide, pH 9.0) over 7 min were used. All samples were resuspended to a concentration of 100 μg/mL in methanol-water (50%:50%).

**(iii) Chemical standard curve creation and calculation.** First, we calculated control curves by measuring the content of six chemical standards for baeocystin, norbaeocystin, norpsilocin, psilocin, psilocybin, and tryptophan at three different concentrations in triplicate (Fig. S2 and Table S3). This allowed us to identify our compounds in spectral data based on retention times and calculate the concentration of each compound in each sample based on extracted *m/z* intensities (Table S4). Standard curves were measured by running standards at 1.0-, 1.2-, and 1.5-μg injections in triplicate and in three replicates. The baseline integrations were recorded for each, and the standard curves were generated using the line of best fit from the standard measurements.

### Cultivation of *P. cubensis*.

Specimen cultivation and processing were carried out as follows. Specimens of *P. cubensis* were cultivated from spore syringes acquired from Spore Works (sporeworks.com; Golden Teacher spore syringe microscopy kit, SKU PSMICsy-090) inoculated on 2% malt agar plates with 50 μg/mL ampicillin antibiotic. Isolated sections of contiguous mycelium from initial spore plates were subcultured by removing ~0.2 cm of tissue from the edge of the growing mycelium with a flame-sterilized scalpel to another plate of 2% malt agar and ampicillin with a thin sheet of sterilized cellophane plastic placed on the surface to facilitate the removal of mycelium for chemical processing. A single culture was used for chemical analysis as well as cultivation of sporocarps. Agar plugs from this culture were placed into spawn growth medium and incubated at room temperature without a consistent light cycle for ~2 to 3 weeks until medium was entirely colonized by mycelium based on visual inspection. Spawn growth was achieved using 1-L wide-mouth Mason jars (Amazon, ASIN: B07HGG3DD1) containing one bag of Ben’s Original Ready Rice whole-grain brown rice (Amazon, ASIN: B00G9U46DW), topped with custom lids that included a 0.22-μm filter for sterile airflow (Amazon, ASIN: B08M5XPFTP), with autoclaving for 40 min on a liquid cycle. To induce sporocarps, fully colonized spawn was mixed aseptically with one brick of coconut coir (Amazon, ASIN: B01M8FUUSJ), 300 g of vermiculite (Amazon, ASIN: B001693Y3Y), and enough tap water (anecdotally thought to be better than deionized [DI] H_2_O) to fill half a container with a moist but not muddy consistency within an 18-qt plastic bin (Amazon, ASIN: B01C3BGTAS).

After 2 to 3 weeks, the lid was left ajar for increased airflow to promote sporocarp production (~1 week). Mycelium and sporocarps underwent lyophilization prior to chemical analysis. Viable culture plugs were retained for long-term storage at room temperature in 50 mL of distilled water (87), and lyophilized voucher samples used for chemistry are deposited in the Garret Herbarium at the Natural History Museum of Utah (UT-M) under the accession numbers listed in Table 2.

### UPLC-MS of cultivated *Psilocybe cubensis* samples.

We applied a UPLC-MS-based untargeted metabolomics pipeline to identify shared and unique metabolites across three cultivated *P. cubensis* sporocarps and vegetative mycelium. Tissue samples were lyophilized, ground, and extracted in 80:20 (vol/vol) methanol-water, producing 2 mL of retained supernatant from 100 mg (±2.5 mg) of sample for spectroscopic analysis. Small molecules (detector range of 50 to 2,000 Da) from the extraction were analyzed using UHPLC (Waters Acquity I-Class, 2.1- by 100-mm BEH amide columns) and mass spectrometry (Waters Xevo G2 quadrupole time of flight [QToF]) (UPLC-MS) in positive and negative ionization mode. Additionally, MS/MS spectra were acquired for sample Psi-6-Cap by running data-dependent acquisition mode (DDA), whereby MS/MS data were collected for all metabolites that were ionized above a set threshold (total ion current [TIC] of 5,000).

### Data processing and spectral feature analysis.

Raw data from the UPLC-MS analysis were processed using the R package XCMS (88) for peak detection, peak alignment, and peak filtering. We used the following parameters: peak detection method ‘*centWave*’ [ppm = 15, peakwidth = c(0.2, 5), snthresh = 5, prefilter = c(1,500)]; peak grouping method ‘*density*’ (bw = 3); and retention time correction method “peakGroups,” in which retention time standards containing eight known compounds as a reference sample and integrate-areas-of-missing-peaks method “FillChromPeaksParam” were used. We then used the R package Camera (89) to assign the various coeluting features (unique *m/z*-retention time combinations) derived from one compound into pc groups. The parameters used were peak grouping after retention time ‘*groupFWHM*’ (perfwhm, 0.7; sigma = 6), verify grouping ‘*groupCorr*’, annotate isotopes ‘*findIsotopes*’, and annotate adducts ‘*findAdducts*’ (polarity = ‘positive’).

Chemical profile comparison was performed by using the intensity values for each identified factor across all cultivated samples. Intensity values were processed and normalized by the total using the decostand command from the R package Vegan (90). Normalized values were then used for PCA and PLS-DA, which were calculated and visualized using the R package mixOmics (91). To investigate the similarity of the chemical profiles, Euclidean distances were calculated using the base R function dist. These calculations were then assigned hierarchical clustering using the base R function hclust. These two measurements were then visualized together using the aheatmap function of the R package NMF (92).

### GNPS annotation to known compounds.

The identification of compounds from metabolic studies is often one of the most difficult parts of chemical profiling, as thousands of compounds and their derivatives are often produced but most lack standard references. In an attempt to make annotation and exploration of metabolomic data sets more accessible, GNPS was created as an initiative to make a standardized and accessible platform to compare spectral data across numerous organisms and chemical databases, as well as providing a platform that would allow older data to be reanalyzed as databases become better over time (50, 93). GNPS utilizes a molecular networking approach that groups sets of spectra from related molecules together to allow for comparison to known compounds even if the specific spectrum being compared has never been identified. In this regard, annotations with high MQscores may have structure and function highly similar to those of the known compounds, while those with lower scores may be similar in structure but have novel or altered function compared to the known compound.

A molecular network was created using the online workflow (https://ccms-ucsd.github.io/GNPSDocumentation/) on the GNPS website (http://gnps.ucsd.edu). The data were filtered by removing all MS/MS fragment ions within ±17 Da of the precursor *m/z*. MS/MS spectra were window filtered by choosing only the top 6 fragment ions in the ±50-Da window throughout the spectrum. The precursor ion mass tolerance was set to 2.0 Da, and the MS/MS fragment ion tolerance was set to 0.5 Da. A network was then created where edges were filtered to have a cosine score above 0.7 and more than 6 matched peaks. Further, edges between two nodes were kept in the network only if each of the nodes appeared in the other’s respective top 10 most similar nodes. Finally, the maximum size of a molecular family was set to 100, and the lowest-scoring edges were removed from them until the molecular family size was below this threshold. The spectra in the network were then searched against the GNPS spectral libraries. The library spectra were filtered in the same manner as the input data. All matches kept between network spectra and library spectra were required to have a score above 0.7 and at least 6 matched peaks.

### Data availability.

All edited ITS sequence data for all specimens were submitted to the National Center for Biotechnology Information with NCBI accession numbers reported in Table S2. The collection database, multiple sequence alignments, and trees are available in Figshare project number 129332. The whole metabolomic spectra have been submitted as a MASSIVE data set available at https://massive.ucsd.edu/ProteoSAFe/private-dataset.jsp?task=1b798991ec6b40d48b49b74e971e2ecf.
