# Supplementary material for: DNA Authentication and Chemical Analysis of Psilocybe Mushrooms Reveal Widespread Misdeterminations in Fungaria and Inconsistencies in Metabolites
Source: Appl Environ Microbiol. 2022 Nov 29;88(24):e01498-22. doi: 10.1128/aem.01498-22 (PMC9764976; doi:10.1128/aem.01498-22)
Supplement: Supplemental file 1 — Fig. S1 to S4. Download aem.01498-22-s0001.pdf, PDF file, 0.5 MB [file aem.01498-22-s0001.pdf]

Supplementary information:

SI-1: Document describing all recognized species of *Psilocybe* before and after 2006 with specimens only known from type collections highlighted. *Psilocybe* s.s. are colored green, those recognized as *Deconica* as red, and those found to be other taxa in purple. Here 141 unique taxonomic names are recognized as *Psilocybe* s.s. However, with future molecular and phylogenetic studies this number is likely to change, with many specimens currently in need of phylogenetic analysis, as well as taxonomic revision.

Supplementary Figures:

S1: Phylogenetic ITS rDNA tree using NCBI and UNITE sequences for *Psilocybe.*

S2: Chemical stack plots and chemical curve formulas.

S3: PCA of chemical factors from cultivated samples.

S4: PLS-DA analysis of cultivated samples.

Table S1: Table of chemical studies and methods of accepted *Psilocybe* spp.

Table S2: Voucher table for all generated ITS barcodes.

Table S3: Chemical control intensity values.

Table S4: Table of chemical factors and intensities.

Table S5: Table of annotated compounds from Psi_6_cap

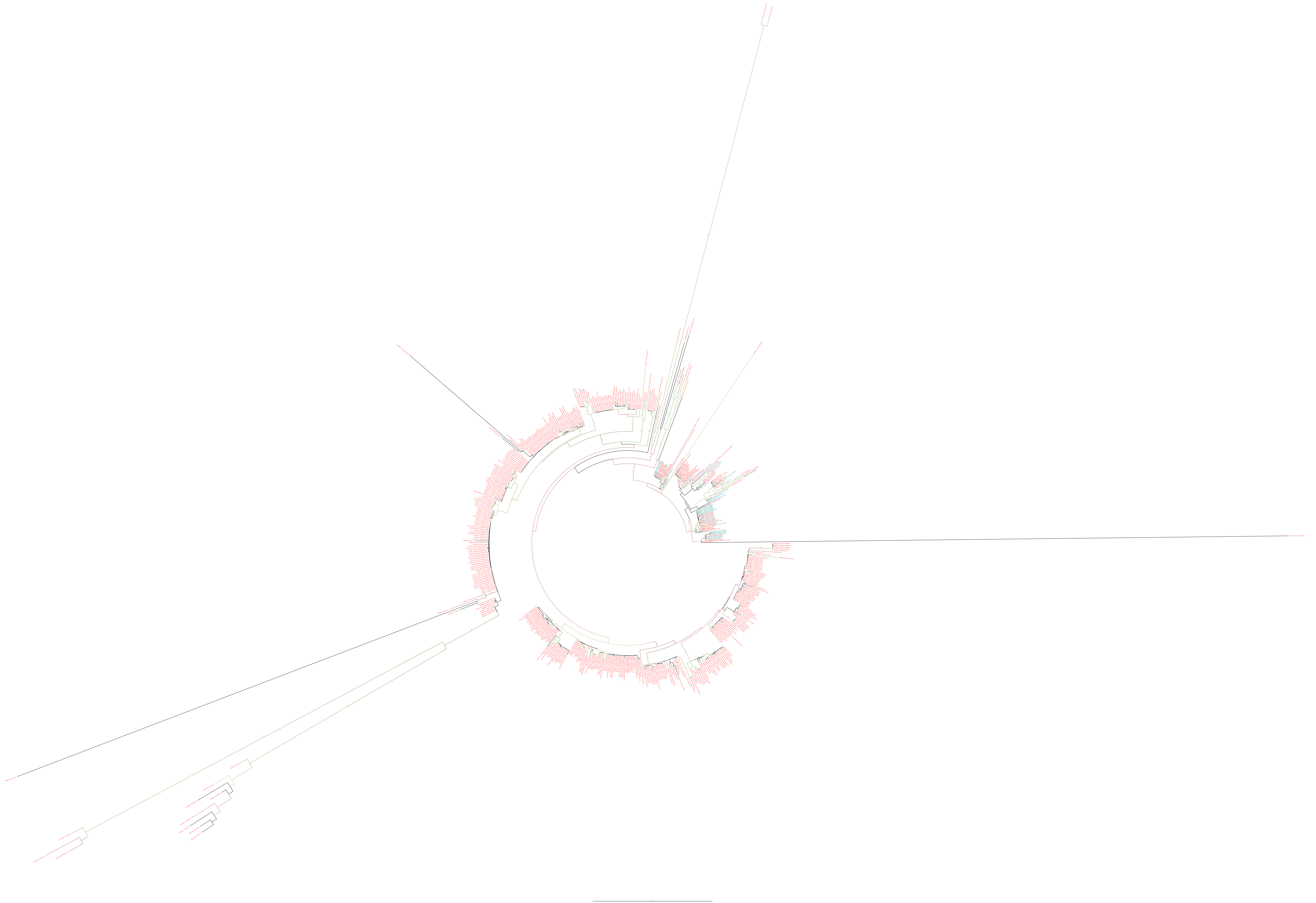

Supplementary figure 2:
Chemical standard trace plot along with all curve formulas and info
(Top) Chemical standard information for each standard including mass concentration, column retention time, compound mass, and slope formula from each standard curve.
(Bottom) chemical standard stack plot showing extracted ion content (EIC) for psilocybin, psilocin and its chemical intermediates produced during natural psilocybin production in *Psilocybe* species.

Chemical Standard Information

Standard	Retention Time	M/Z	Concentration	Curve Formula	R^2
Tryptophan	4.657'	205	100 ug/mL	y = 1E+06x	1.00
Psilocin	1.86'	205	100 ug/mL	y = 8E+06x	1.00
Psilocybin	6.087'	285	100 ug/mL	y = 1E+07x	0.99
Baeocystin	6.23'	271	100 ug/mL	y = 9E+06x	0.98
Norbaeocystin	6.412'	257	100 ug/mL	y = 1E+06x	1.00
Bufotenine	3.3'	205	100 ug/mL	y = 5E+06x	1.00
Norpsilocin	3.7'	191	100 ug/mL	y = 3E+06x	0.99

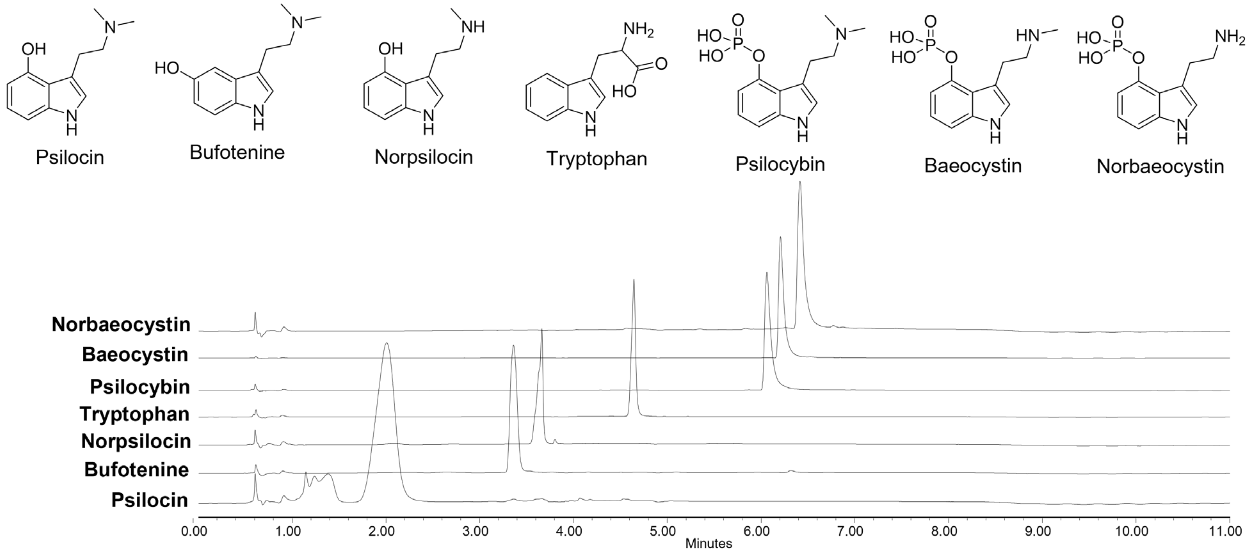

Supplementary figure 3:
Principal component analysis of the intensity for each identified chemical factor. Arrows indicate positive correlation based on samples. Most factors overlap between samples; however a general pattern of correlation occurs between caps and steps (Psi_5, and Psi_6), but separates between growing bins (Psi_4 vs Psi_5 and Psi_6)

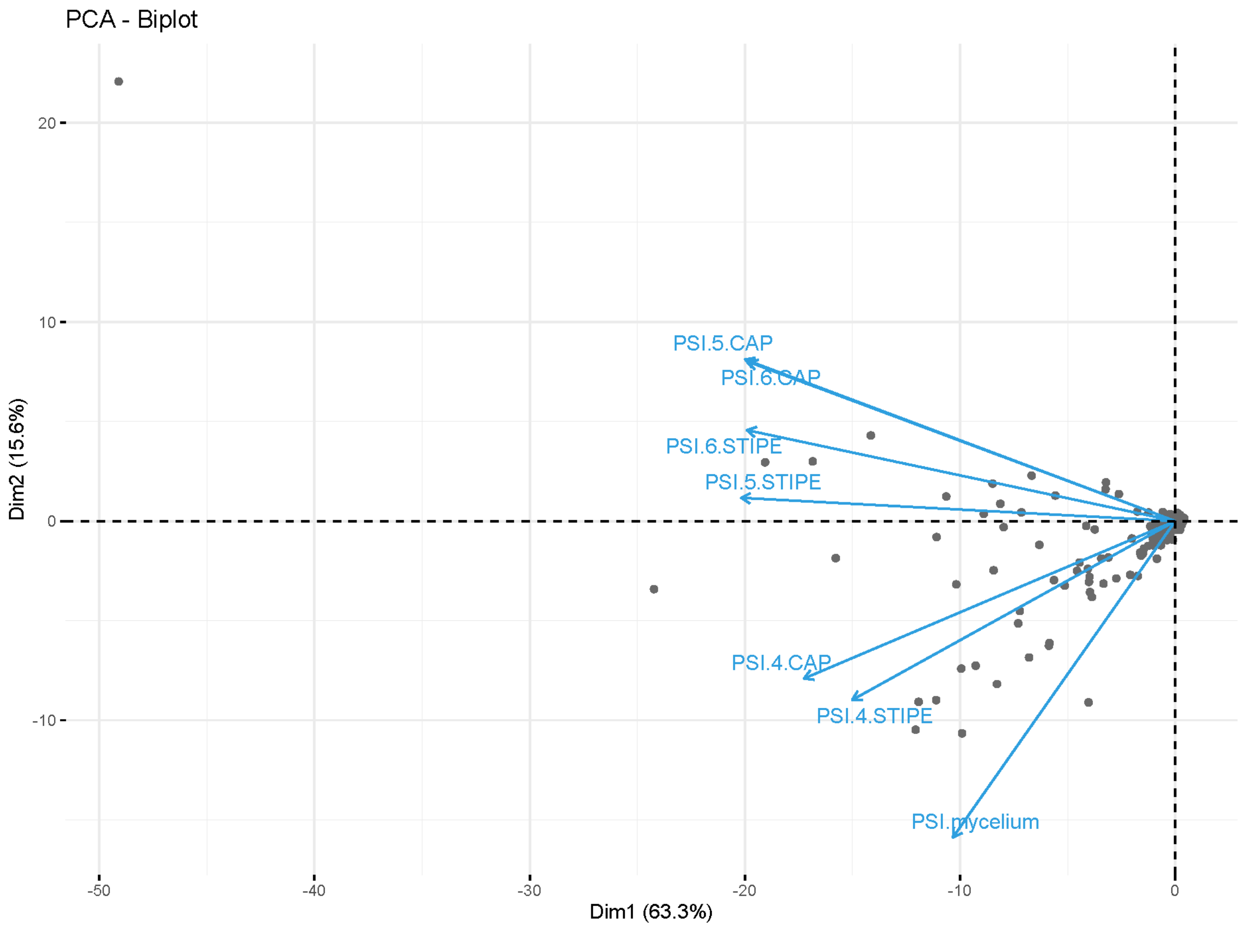

Supplementary figure 4:
PLS-DA of samples based on chemical factors. A general pattern of correlation occurs between caps and steps (Psi_5, and Psi_6), but separates between growing bins (Psi_4 vs Psi_5 and Psi_6)

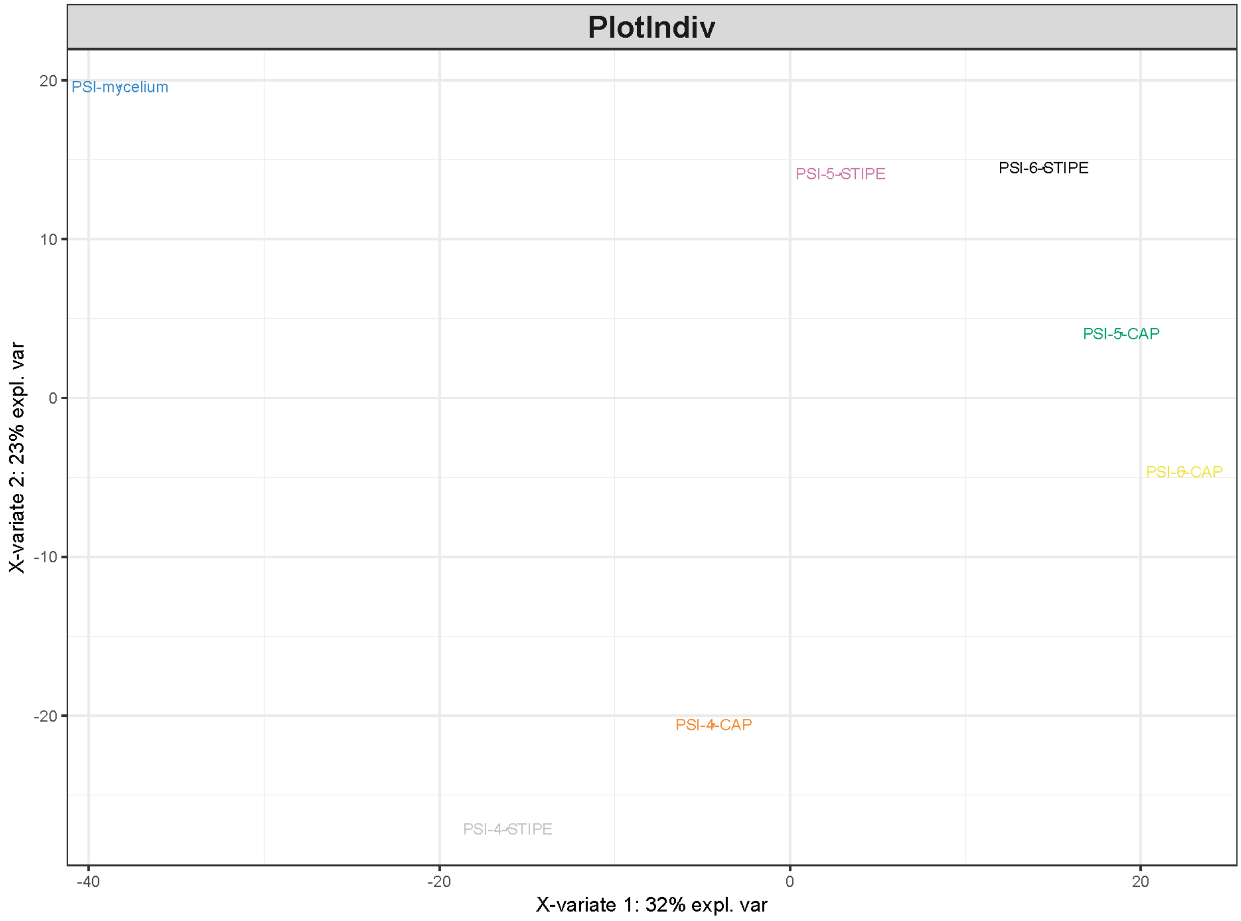

1. Repke DB, Leslie D, Guzmán G. Baeocystin in psilocybe, conocybe and panaeolus. Lloydia. 1977;

2. Wurst M, Kysilka R, Koza T. Analysis and isolation of indole alkaloids of fungi by high-performance liquid chromatography. Journal of Chromatography A. 1992 Feb;593(1–2):201–8.

3. Wurst M, Semerdžieva M, Vokoun J. Analysis of psychotropic compounds in fungi of the genus psilocybe by reversed-phase high-performance liquid chromatography. Journal of Chromatography A. 1984 Mar;286:229–35.

4. Borovička J, Oborník M, Stříbrný J, Noordeloos ME, Sánchez LAP, Gryndler M. Phylogenetic and chemical studies in the potential psychotropic species complex of <I>Psilocybe atrobrunnea</I> with taxonomic and nomenclatural notes. Pers - Int Mycol J. 2015 Jun 29;34(1):1–9.

5. Leung AY, Smith AH, Paul AG. Production of Psilocybin in Psilocybe baeocystis Saprophytic Culture. Journal of Pharmaceutical Sciences. 1965 Nov;54(11):1576–9.

6. Stijve T, Kuyper Th. Occurrence of Psilocybin in Various Higher Fungi from Several European Countries. Planta Med. 1985 Oct;51(05):385–7.

7. Stríbrný J, Borovička J, Sokol M. Levels of psilocybin and psilocin in various types of mushrooms. Soudní lékarství / casopis Sekce soudního lékarstvi Cs lékarské spolecnosti J Ev Purkyne. 2003 Aug 1;48:45–9.

8. Borovička J. New variety of Psilocybe moravica and notes on Psilocybe bohemica. Czech Mycology. 2006 Jan 1;58:75–80.

9. Ohenoja E, Jokiranta J, Mäkinen T, Kaikkonen A, Airaksinen MM. The Occurrence of Psilocybin and Psilocin in Finnish Fungi. J Nat Prod. 1987 Jul;50(4):741–4.

10. Pedersen-Bjergaard S, Rasmussen KE, Sannes E. Strategies for the capillary electrophoretic separation of indole alkaloids inPsilocybe semilanceata. Electrophoresis. 1998 Jan;19(1):27–30.

11. Repke DB, Leslie DT. Baeocystin in Psilocybe semilanceata. Journal of Pharmaceutical Sciences. 1977 Jan;66(1):113–4.

12. Borner S, Brenneisen R. Determination of tryptamine derivatives in hallucinogenic mushrooms using high-performance liquid chromatography with photodiode array detection. Journal of Chromatography A. 1987 Jan;408:402–8.

13. Christiansen AL, Rasmussen KE, Tønnesen F. Determination of psilocybin in Psilocybe semilanceata using high-performance liquid chromatography on a silica column. Journal of Chromatography A. 1981 May;210(1):163–7.

14. Guzmán G, Ott J. Description and Chemical Analysis of a New Species of Hallucinogenic *Psilocybe* from the Pacific Northwest. Mycologia. 1976 Nov;68(6):1261–7.

15. Margot P, Watling R. Studies in Australian Agarics and Boletes. Transactions of the British Mycological Society. 1981 Jun;76(3):485–9.

16. Beug M, Stamets P, Bigwood J, Guzman G. A New Species and a New Variety of Psilocybe from North America. Mycotaxon. 1980 Jul 1;XI:476–84.

17. Heim R, Hofmann A. Isolement de la psilocybine à partir du Stropharia cubensis Earle et d'autres espèces de champignons hallucinogènes mexicains appartenant au genre Psilocybe. Paris, France: Gauthier-Villars]; 1958. 5 p.

18. Ola'h GM, Heim R. [A new North American species of Psilocybe hallucinogen: Psilocybe quebecensis G. Ola'h and R. Heim]. C R Acad Hebd Seances Acad Sci D. 1967 Mar 20;264(12):1601–4.

19. Tsujikawa K, Kanamori T, Iwata Y, Ohmae Y, Sugita R, Inoue H, et al. Morphological and chemical analysis of magic mushrooms in Japan. Forensic Science International. 2003 Dec;138(1–3):85–90.

20. Anastos N, Barnett N, Lewis S, Gathergood N, Scammells P, Sims D. Determination of psilocin and psilocybin using flow injection analysis with acidic potassium permanganate and tris(2,2'-bipyridyl)ruthenium(II) chemiluminescence detection respectively. Talanta. 2005 Aug 15;67(2):354–9.

21. Guzmán G. The genus Psilocybe: a systematic revision of the known species including the history, distribution, and chemistry of the hallucinogenic species. Vaduz [Liechtenstein]: J. Cramer; 1983. 439 p. (Beihefte zur Nova Hedwigia).

22. Picker J, Rickards R. The occurrence of the psychotomimetic agent psilocybin in an Australian agaric, Psilocybe subaeruginosa. Aust J Chem. 1970;23(4):853.

23. Vanhaelen-Fastré R, Vanhaelen M. Qualitative and quantitative determinations of hallucinogenic components of psilocybe mushrooms by reversed-phase high-performance liquid chromatography. Journal of Chromatography A. 1984 Jan;312:467–72.

24. White PC. Analysis of extracts from Psilocybe semilanceata mushrooms by high-pressure liquid chromatography. Journal of Chromatography A. 1979 Feb;169:453–6.

25. Gartz J, Allen JW, Merlin MD. Ethnomycology, biochemistry, and cultivation of Psilocybe samuiensis Guzmán, Bandala and Allen, a new psychoactive fungus from Koh Samui, Thailand. Journal of Ethnopharmacology. 1994 Jul;43(2):73–80.

26. Guzmán G, Jacobs J, Ramírez F, Murrieta-Hernández D, Gándara E. The taxonomy of Psilocybe fagicola-complex. Journal of microbiology (Seoul, Korea). 2005 Apr 1;43:158–65.

27. Keller T, Schneider A, Regenscheit P, Richard Dirnhofer, Rücker T, Jaspers J, et al. Analysis of psilocybin and psilocin in Psilocybe subcubensis GUZMÁN by ion mobility spectrometry and gas chromatography–mass spectrometry. Forensic Science International. 1999 Jan;99(2):93–105.

28. Mantle PG, Waight ES. Occurrence of psilocybin in the sporophores of Psilocybe semilanceata. Transactions of the British Mycological Society. 1969 Oct;53(2):302–4.

29. Allen J, Gartz, Molter D, Sihanonth P. The Occurrence, Cultivation, and Chemistry of Psilocybe ovoideocystidiata, a new Bluing Species (Agaricales) from Ohio, Pennsylvania and West Virginia By

Allen, John W., Gartz, Jochen., Molter, Dan, and Prakitsin Sihanonth. Ethnomycological Journals: Sacred Mushroom Studies. 2009 Dec 1;75–86.

30. Beck O, Helander A, Karlson-Stiber C, Stephansson N. Presence of Phenylethylamine in Hallucinogenic Psilocybe Mushroom: Possible Role in Adverse Reactions. Journal of Analytical Toxicology. 1998 Jan 1;22(1):45–9.

31. Christiansen A, Rasmussen K, Høiland K. The Content of Psilocybin in Norwegian Psilocybe semilanceata. Planta Med. 1981 Jul;42(07):229–35.

32. Heim R. Les champignons toxiques et hallucinogènes. 2. éd. entièrement rev. et augm. Paris: Boubée; 1978. 270 p. (Collection Faunes et flores actuelles).

33. Nkadimeng SM, Nabatanzi A, Steinmann CML, Eloff JN. Phytochemical, Cytotoxicity, Antioxidant and Anti-Inflammatory Effects of Psilocybe Natalensis Magic Mushroom. Plants (Basel). 2020 Aug 31;9(9):E1127.

34. Semerdžieva M, Wurst M, Koza T, Gartz J. Psilocybin in Fruchtkörpern von *Inocybe aeruginascens*. Planta Med. 1986 Apr;52(02):83–5.

35. Hofmann A, Heim R, Brack A, Kobel H. Psilocybin, ein psychotroper Wirkstoff aus dem mexikanischen RauschpilzPsilocybe mexicana Heim. Experientia. 1958 Mar;14(3):107–9.

36. Ainsworth GC, Bisby GR, Kirk PM. Ainsworth & Bisby's dictionary of the fungi / by P.M. Kirk ... [et al.] ; with the assistance of T.V. Andrianova ... [et al.]. 10th ed. Wallingford, Oxon, UK: CABI; 2008. 771 p.

37. Benedict RG, Tyler VE, Watling R. BLUEING IN CONOCYBE PSILOCYBE AND A STROPHARIA SPECIES AND DETECTION OF PSILOCYBIN. Lloydia. 1967;30(2):150.

38. Froese T, Guzmán G, Guzmán-Dávalos L. On the Origin of the Genus Psilocybe and Its Potential Ritual Use in Ancient Africa and Europe1. Econ Bot. 2016 Jun;70(2):103–14.

39. Gartz J. Cultivation and analysis of Psilocybe species and an investigation of Galerina steglichii. Rovereto: Musei Civici di Rovereto; 1994.

40. Guzmán G, Allen J, Gartz J. A Worldwide geographical distribution of the Neurotropic Fungi, an analysis and discussion. Ann Mus Civ Rovereto. 1998 Jan 1;14.

41. Heim R, Wasson RG. Les champignons hallucinogènes du Mexique: ètudes ethnologiques, taxinomiques, biologiques, physiologiques et chimiques. Paris: Mus??um National d'Histoire Naturelle; 1959.

42. Ma T, Feng Y, Lin XF, Karunarathna SC, Ding WF, Hyde KD. Psilocybe chuxiongensis, a new bluing species from subtropical China. Phytotaxa. 2014 Jan 21;156(4):211.

43. Moncalvo JM, Vilgalys R, Redhead SA, Johnson JE, James TY, Catherine Aime M, et al. One hundred and seventeen clades of euagarics. Molecular Phylogenetics and Evolution. 2002 Jun;23(3):357–400.

44. Moser M, Horak E. Psilocybe Serbica Spec. Nov: Eine Neue Psilocybin und Psilocin Bildende Art Aus Serbien. 1968.

45. Norvell LL. Report of the Nomenclature Committee for Fungi: 15. McNeill J, Redhead SA, Wiersema JH, editors. Taxon. 2010 Feb;59(1):291–3.

46. Semerdzieva M. Halluzinogene Pilze in der Tschechoslowakei. 1973;

47. Stamets P, Gartz J. A new caerulescent Psilocybe from the Pacific Coast of Northwestern America. Integration. 1995;6:21–8.

48. Stijve T, De Meijer AAR. Macromycetes from the State of Parana, Brazil. 4. The Psychoactive Species. ARQUIVOS DE BIOLOGIA E TECNOLOGIA. 1993;36(2):313.

49. Tyler VE. Indole derivatives in certain North American mushrooms. Lloydia. 1961;24(2):71.
